# Impact of social support on college students’ anxiety due to COVID-19 isolation: Mediating roles of perceived risk and resilience in the postpandemic period

**DOI:** 10.3389/fpsyg.2022.948214

**Published:** 2022-11-24

**Authors:** Tai Bo He, Chia Ching Tu, Xue Bai

**Affiliations:** ^1^Education Science College, Weinan Normal University, Weinan, China; ^2^International College, Krirk University, Bangkok, Thailand; ^3^Foreign Language Middle School, Xinzhou Teachers University, Xinzhou, China

**Keywords:** anxiety, COVID-19, perceived risk, postpandemic, resilience, social support

## Abstract

**Introduction:**

Because of the outbreak of COVID-19, several colleges and universities in Xi’an, China, implemented quarantine measures and closed their gates, which increased anxiety among the students.

**Methods:**

The Perceived Social Support Scale, Perceived Risk of COVID-19 Pandemic Scale, Connor–Davidson Resilience Scale, and Self-Rating Anxiety Scale were used for measurements. SPSS26 and AMOS26 (IBM SPSS AMOS Statistics, New York, United States) were used for data analysis. Structural equation modeling (SEM) was used to test the data from the 2,251 respondents and the hypothetical model.

**Results:**

The students’ perceived social support was discovered to have had a significant negative effect on anxiety. The students’ perceived COVID-19 risk and resilience played significant mediating roles in the relationship between social support and anxiety.

**Discussion:**

When college students feel social support and have resilience, they can reduce anxiety during the epidemic stage. Therefore, education administrators and parents should help college students to identify the current situation of the epidemic environment, enrich relevant knowledge.

## Introduction

COVID-19 was identified as a pandemic by the World Health Organization (WHO) on 11 March 2020 (WHO, 2020). It has affected people’s mental health, inducing fear, anxiety, distress, and isolation (Qiu et al., 2020; Rajkumar, 2020). Globally—as of 5:44 p.m. CET, 17 February 2022—416,614,051 confirmed COVID-19 cases, including 5,844,097 deaths, have been reported to the WHO (WHO, 2022).

In December 2021, a severe COVID-19 outbreak occurred in Xi’an. Some community infection cases were reported within the university campus. Accordingly, the Xi’an government took emergency measures to prevent the spread of the virus and maximize safety. The surrounding universities and communities responded by closing their schools and isolating at home, respectively. Nearly 2 million college students had to stay in their dormitories under quarantine, which severely affected their lives and learning (Xi'an Municipal Government, 2021).

The global COVID-19 pandemic has increased people’s stress (Lee and Crunk, 2020), and the sense of fear among individuals, including college students, has been considerably higher than that during other disease outbreaks (Rubin and Wessely, 2020; Shigemura et al., 2020; Haikalis et al., 2022). Among college students, coursework, stress levels, and perceived mental health have been affected to some extent. Studies have shown that stress caused by the COVID-19 pandemic has influenced a high proportion of college students (Haikalis et al., 2022).

The incidence of anxiety and depressive symptoms in college students became a major problem even before the outbreak of COVID-19. The World Health Organization World Mental Health International College Student (WMH-ICS) surveyed a sample of more than 200,000 college students in 28 countries and found that college is a critical period for human intellectual development, but also the peak period for mental illness (Cuijpers et al., 2019). Studies have shown that more than 20 percent of college students in China suffer from psychiatric disorders, and this rate has been growing (Liu et al., 2019a,b; Gao et al., 2020). Loneliness because of COVID-19-related isolation and social distancing has negatively affected individuals’ mental health, anxiety, and perceived social support, thus reducing their sense of happiness and increasing their risk of mental health problems; these outcomes of lockdown and quarantining are prevalent, particularly among older people and college students (Cipolletta et al., 2021; Cipolletta and Gris, 2021; Ernst et al., 2022).

Studies have also suggested that experiencing stress and anxiety are significant indicators of fear induced by COVID-19 (Asmundson and Taylor, 2020; Lee and Crunk, 2020; Ornell et al., 2020). College students are considered more susceptible to mental health problems than their non-college-attending peers (Cvetkovski et al., 2012; Barnett et al., 2019). During the outbreak of COVID-19, increases have been observed in the incidence of various mental health problems of college students; these problems include anxiety, perceived stress, and depression (Huang and Zhao, 2020; Odriozola-González et al., 2020; Sundarasen et al., 2020). The incidence rate of anxiety has been reported to be >28.8% among students (Chang et al., 2020; Wang et al., 2020). Anxiety is likely to cause poor academic performance and academic failure among college students, resulting in the continuous escalation of mental health complications and possibly even suicide attempts (Craske et al., 2017).

Studies have indicated that individual resilience is neither innate nor stable but is variable (Ward et al., 2011; Lunnay et al., 2021). The resilience–stress theory suggests that when individuals are stressed or experience challenging events in daily life, a resilience mode is activated to help them cope with their stress (Fletcher and Sarkar, 2013).

Some scholars believe that social support, resilience, and perceived stress are significantly related to anxiety among college students in the face of the COVID-19 epidemic. Social support can negatively affect the stress perceived by an individual, positively affect their resilience, and negatively affect their anxiety level to a certain extent (Muyor-Rodríguez et al., 2021). However, research on (1) the interactions among these four variables including the combined impact of these factors as well as the impact of only social support on anxiety among college students under perceived pressure during isolation and (2) the role of an individual’s resilience during the COVID-19 epidemic is lacking.

Therefore, based on the above research gaps, exploring the roles of and correlations among social support, COVID-19 risk perception, resilience, and anxiety among college students during COVID-19 isolation is of great importance.

## Literature review

Social support is a type of information that individuals acquire and perceive from their external environment including their social network, and it enables individuals to feel care and love from others (Rosenbaum, 2006). Individuals who are isolated during the COVID-19 pandemic may feel lonely because of a lack of opportunities to receive face-to-face social support from the outside world (Ernst et al., 2022).

Social support is negatively correlated with anxiety level (Ao et al., 2020; Grey et al., 2020). The overall anxiety level of Chinese college students was higher than that of other groups during the COVID-19 epidemic (Wang and Zhao, 2020), and college students who perceived themselves as having lower social support were more likely to have anxiety symptoms (Ma et al., 2020). This study therefore proposed the following hypothesis:

*H1*: College students’ perceptions of social support negatively affect their COVID-19 anxiety during school closure and isolation.

The perceived risk posed by the epidemic is the risk and stress felt by individuals during COVID-19, a stressor (Li and Lyu, 2021). Studies have shown that when faced with external stresses and limited resources brought about by the epidemic, individuals may perceive greater pressure in the presence of external stress and limited resources resulting from the epidemic (Karkoulian et al., 2016). Individuals with higher social support exhibit greater resilience, which contributes to stress management (Kalaitzaki et al., 2020). Because a certain correlation exists between social support and perceived stress, social support can alleviate the adverse reactions caused by such stress and reduce its level; thus, social support negatively affects perceived stress (Beehr et al., 2010). Studies have reported that the perceived risk posed by the COVID-19 epidemic has been a crucial contributor to anxiety (Rajkumar, 2020). Scholars have also stated that social support can weaken the negative effects of stress on anxiety; that is, social support negatively affects perceived stress and anxiety (Beehr et al., 2010; Thoits, 2011).

Therefore, the second hypothesis was proposed:

*H2*: The risk perceived by college students regarding the epidemic plays a mediating role in the relationship between their sense of social support and level of anxiety.

Resilience refers to the ability to rebound when facing stress or frustration (Leary et al., 1999). This ability determines whether an individual can effectively cope with major pressures such as setbacks and difficulties. As a vital predictor of a person’s mental health level (Rosenberg, 1965), resilience negatively affects anxiety (Smith et al., 2008). Studies have found that resilience may directly affect an individuals’ dynamic regulation and adaptability (White et al., 2010) and that resilience has significant negative correlations with negative emotions such as depression and anxiety, suggesting a critical role of resilience in maintaining good mental health (Reivich et al., 2011; Tuck and Andersin, 2014; Gheshlagh et al., 2017; Johnson et al., 2017).

Given that social adaptation is a basic task in the course of life, the level of social adaptability of an individual is usually expressed in terms of the social adaptability quotient. Individuals with high emotional intelligence tend to exhibit relatively high social adaptability (Vaillant and Davis, 2010) because emotional intelligence is a core indicator of resilience (Armstrong et al., 2011). Therefore, resilience is strongly correlated with social adaptability (Patricia et al., 2015; Gheshlagh et al., 2017), which affects the ability of college students to adapt to the adverse situations resulting from isolation and school closures during the COVID-19 pandemic.

Highly resilient college students actively deal with stress and adapt positively to pressure (Yi et al., 2005; Campbell-Sills et al., 2006; Hartley, 2008; Sheard, 2009; Yildirim and Tanriverdi, 2020). The process model of resilience holds that individuals may use various resources to maintain their physical and mental balance during stressful events and that the use of resources such as social support depends on the individuals’ psychological traits (Richardson, 2002).

Thus, if college students receive greater social support during quarantine, their resilience is greater, and their level of epidemic-induced anxiety is lower.

Therefore, the third hypothesis was proposed:

*H3*: College students’ resilience has a mediating effect on the relationship between their perceived social support and anxiety.

## Materials and methods

**Figure 1:**
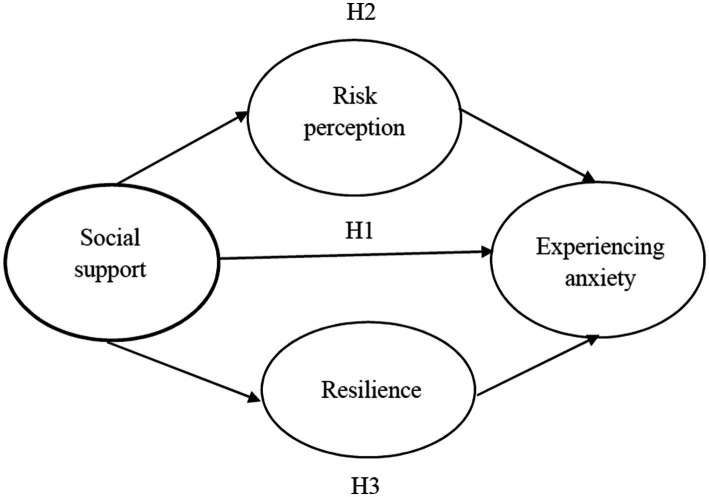
Hypothetical model.

A hypothetical model was proposed (Figure 1) in line with the theoretical basis, purpose, and literature review of the study. Social support was proposed as a positive factor in anxiety, whereas COVID-19 risk perception and resilience were assumed to be two mediators.

### Research process and participants

After obtaining approval from the Scientific Research Ethics Committee of W University (approval number: 2021–1,228-01) and informed consent from the respondents, the study questionnaire was distributed online from January 5 to 12, 2022, through the professional survey platform Questionnaire Star^1^; thus, purposive sampling was performed. The questionnaire comprised a total of 58 questions, including 8 questions regarding the respondents’ demographics, such as their age, sex, and family’s financial status. The questionnaire survey was administered online among students (age = 18–24, mean age = 19.60, SD = 1.299 years) affected by the COVID-19 epidemic in five Xi’an universities and colleges, which had been closed and had imposed quarantine for approximately 7 days. A total of 2,400 questionnaires were distributed. After excluding missing answers and answer patterns demonstrating repetitiveness, 2,251 college students were finally selected as the research participants. The effectiveness rate of the questionnaire was 93.8%. Among the participants, 1,315 were men (58.4%) and 936 were women (41.6%). In terms of educational level, 952 participants were freshmen, 921 were sophomores, 303 were juniors, and 75 were seniors. Overall, 19.2% of the respondents were from a city and 78.3% were from a rural area. The sample data are shown in Table 1.

**Table 1:** Demographic sample.

Basic situation	Category	Frequency	Percentage (%)
Gender	Male	1,315	58.4
	Female	936	41.6
Region	City	432	19.2
	Suburb	56	2.5
	Rural area	1,763	78.3
Grade level	Freshman	952	42.3
	Sophomore	921	40.9
	Junior	303	13.5
	Senior	75	3.3

### Research tools

#### Social support

The Chinese version of the Perceived Social Support Scale, revised by Zimet and Farley (Zimet et al., 1990), is used to analyze social support on the basis of three dimensions: support from family (e.g., “I get emotional help and assistance from my family when I need it”), support from friends (e.g., “When difficulties emerge, I can count on my friends”), and support from other parties (e.g., “When I am in trouble, the comfort from teachers is really helpful”). The scale has four questions related to each dimension, with 12 questions in total. A 5-point Likert scale was employed for scoring, ranging from 1 (*strongly disagree*) to 5 (*strongly agree*). The higher the score, the higher the social support level. The composite reliability (CR) was 0.925, and the average variance extracted (AVE) was 0.848, which indicated high convergence validity of the potential variables (Hair et al., 2006). Each fit index was favorable: χ^2^/df = 4.253 (Schumacker and Lomax, 2004), comparative fit index (CFI) = 0.916, goodness-of-fit index (GFI) = 0.932, and root mean square error of approximation (RMSEA) = 0.038 (McDonald and Ho, 2002; Schumacker and Lomax, 2004; Steiger, 1989). Additionally, the Cronbach’s α was.89, 0.95, and.96 for the three dimensions.

#### COVID-19 risk perception

The Perceived Risk of COVID-19 Pandemic Scale, compiled by Chinese scholars Xi Juzhe and She Zhuang (Li and Lyu, 2021), is used to examine three dimensions: emotional feelings (e.g., “I am worried about contracting the new coronavirus”), cognitive judgments (e.g., “No matter how small the odds, I will contract COVID-19”), and unusual severity characteristic side (e.g., “I often assume what can be done if I’m infected with COVID-19”). The scale has three questions related to each dimension, with a total of nine questions. A 5-point Likert scale was employed for scoring, ranging from 1 (*strongly disagree*) to 5 (*strongly agree*). The higher the score, the greater the perceived COVID-19 risk. The CR was 0.944 and AVE was 0.848, indicating good convergence validity of the potential variables (Hair et al., 2006). Each fit index was favorable: *χ*^2^/df = 2.015 (Schumacker and Lomax, 2004), GFI = 0.997, and RMSEA =0.021 (McDonald and Ho, 2002; Schumacker and Lomax, 2004; Steiger, 1989). The Cronbach’s α = 0.80, 0.81, and.83 for the three dimensions.

#### Resilience

Resilience allows an individual to adapt to pressure and deal with trauma (Campbell-Sills and Stein, 2007). The resilience scale is employed to measure a person’s self-cognition under stressful events. The scale contains 10 items linked to a person’s level of capacity in coping with stress and trauma (Yang et al., 2020). A 5-point Likert scale was employed for scoring, ranging from 1 (*strongly disagree*) to 5 (*strongly agree*). The higher the score, the greater the respondent’s resilience. The CR value was 0.977, and the AVE value was 0.808; these values indicated good convergent validity of the potential variables (Hair et al., 2006). Each fit index was favorable: χ^2^/df = 3.015 (Schumacker and Lomax, 2004), GFI = 0.987, and RMSEA = 0.041 (McDonald and Ho, 2002; Schumacker and Lomax, 2004; Steiger, 1989); the Cronbach’s alpha value was.906.

#### Anxiety

The Self-Rating Anxiety Scale developed by Zung (1971) consists of 20 items divided into four dimensions: experiencing anxiety, with 5 items (e.g., “I feel worried for no reason”); neurological dysfunction, with 5 items (e.g., “I feel like I’m going to pass out”); motor senses tension, 5 items (e.g., “I feel my heart beating faster”); and mixed representation of neurological dysfunction and motor tension, 5 items (e.g., “I do not sleep well at night, and I’m prone to insomnia”; Olatunji et al., 2006). A 4-point Likert scale was employed for scoring, from 1 (complete nonconformity) and 4 (complete conformity). The higher the total score, the greater the anxiety of the individual. The CR was 0.963 and AVE was 0.867, indicating favorable convergence validity of the potential variables (Hair et al., 2006). Each fit index was good: *χ*^2^/df = 4.942 (Schumacker and Lomax, 2004), GFI =0.854, and RMSEA =0.052 (Hu and Bentler, 1999). The Cronbach’s α was.92, 0.92, 0.87, and.83 for the four dimensions.

### Data analysis

SPSS26 and AMOS26 (IBM SPSS AMOS Statistics, New York, United States) were used for data analysis. Structural equation modeling (SEM) was used to test the data from the 2,251 respondents and the hypothetical model. The measurement and structural models were verified (Bollen, 1989; Kline, 2005); therefore, a reasonable measurement model was tested using confirmatory factor analysis. Model parameters and fit indices obtained through maximum likelihood estimation were considered statistical indicators to confirm the degree of collocation between the 2,251 students’ data and the measurement model.

## Results

### Main effect

**Figure 2:**
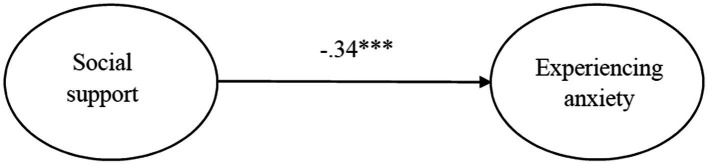
Main effect of social support to experiencing anxiety. *** *p* < 0.001.

When the main effect path was tested using SEM, the standardized regression coefficients of the main effect were between.67 and.96. The model demonstrated by the main effect path was suitable for the sample data: *χ*^2^ = 24.329, *χ*^2^/*df* = 2.027 (Schumacker and Lomax, 2004), RMSEA = 0.021 (McDonald and Ho, 2002; Schumacker and Lomax, 2004; Steiger, 1989), CFI = 0.993 and NFI = 0.986 (Bentler and Bonett, 1980; Hu and Bentler, 1999), GFI = 0.993 (Doll et al., 1994), TLI = 0.988 (Doll et al., 1994; Schumacker and Lomax, 2004), and SRMR = 0.012 (<0.05; Jöreskog and Sörbom, 1989; Hu and Bentler, 1999). Social support explained 43.6% of the variance in experiencing anxiety levels (γ = −0.34, *p* < 0.001), thus supporting Hypothesis 1 (Figure 2).

### Structural model

The standardized regression coefficients of the main effect were between.68 and.95 in the SEM. The model demonstrated by the main effect path was a fit for the sample data: χ^2^ = 294.194 and χ^2^/df = 4.903 (Schumacker and Lomax, 2004), RMSEA = 0.042(<0.05; Browne et al., 1990; McDonald and Ho, 2002; Schumacker and Lomax, 2004; Steiger, 1989), CFI = 0.908 and NFI = 0.888 (Hu and Bentler, 1999), GFI = 0.942 (Doll et al., 1994), TLI = 0.880 (Doll et al., 1994), and SRMR = 0.030 (<0.05; Jöreskog and Sörbom, 1989; Hu and Bentler, 1999).

**Figure 3:**
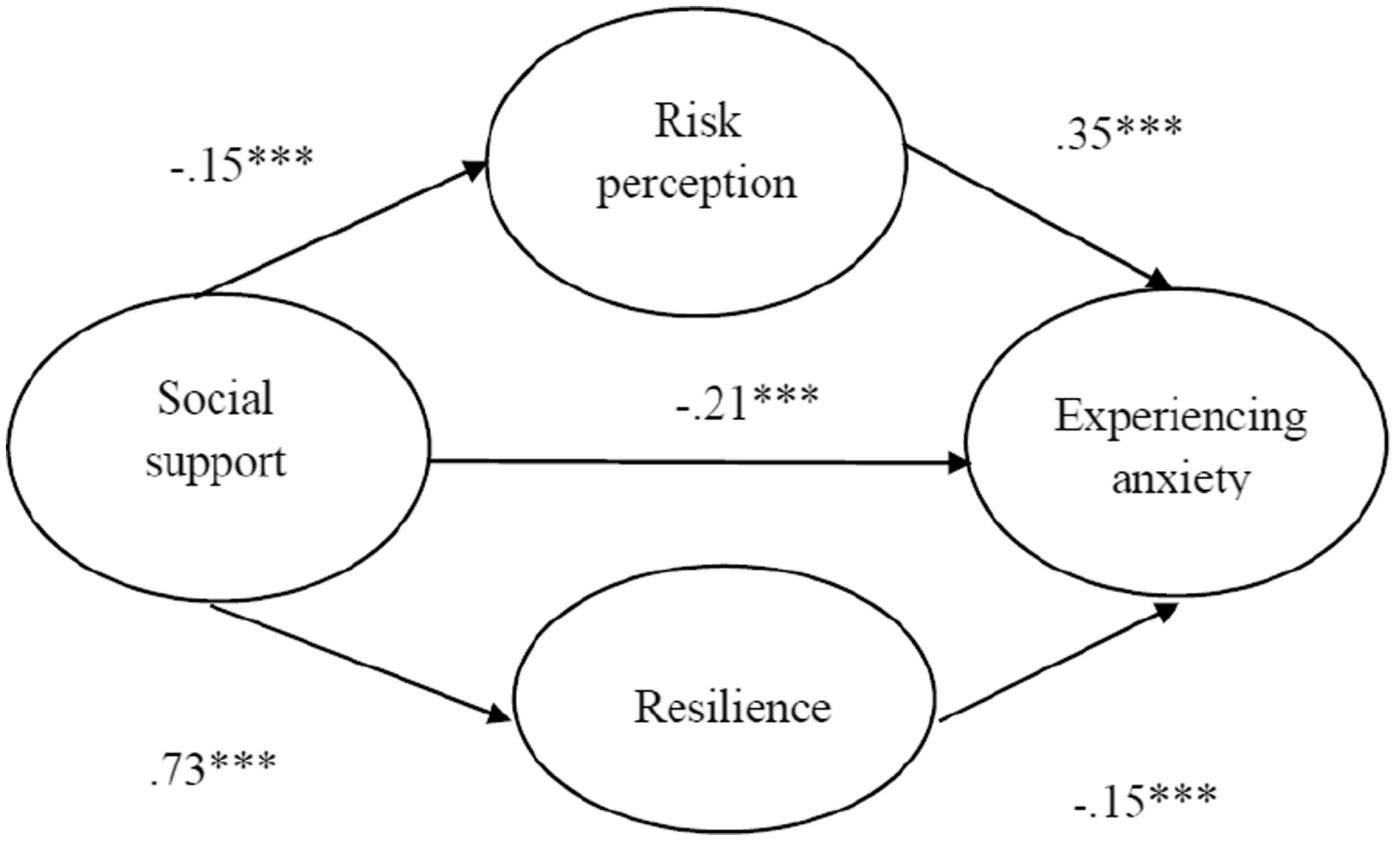
Structural model.

Hypotheses 2 and 3, which involved mediating factors, constituted a structural model, as illustrated in Figure 3.

### Mediating effect

To validate the hypotheses, bootstrapping was employed to conduct regression analysis of the pathways among social support, perceived risk posed by the COVID-19 epidemic, and resilience (Hayes, 2009). Table 2 presents the results of the analysis with bootstrap 95% confidence intervals (1,000 times) all excluding 0 (Mackinnon et al., 2004); the direct impact of social support on experiencing anxiety was significant (γ = −0.229, *p* < 0.001). For the social support → C19RP → experiencing anxiety path, the bootstrap 95% confidence intervals (1,000 times) all excluded 0 (γ = −0.031, *p* < 0.001), with the mediating effect found to be significant. For the social support → RE → experiencing anxiety path, the bootstrap 95% confidence intervals (1,000 times) again all excluded 0 (γ = −0.197, *p* < 0.001), with the mediating effect discovered to be significant.

**Table 2:** Mediating effects test.

Total, direct, and indirect effect	Point estimate	Product of coefficient		Bootstrapping 1,000 times 95CI			
				Bias-corrected		Percentile	
		SE	Z	Lower	Upper	Lower	Upper
SS → C19RP → EA	−0.031	0.013	−2.385	−0.058	−0.006	−0.057	−0.005
SS → RE → EA	−0.197	0.028	−7.036	−0.252	−0.146	−0.253	−0.147
Direct effect:SS → EA	−0.229	0.040	−5.725	−0.304	−0.148	−0.303	−0.148
Total indirect effect	−0.229	0.031	−7.387	−0.291	−0.169	−0.292	−0.170
Total effect	−0.457	0.035	−13.057	−0.527	−0.389	−0.529	−0.391

SS, Social Support; C19RP, COVID-19Risk perception; RE, Resilience; EA, Experiencing anxiety.

## Discussion

### Effects of social support on the level of experiencing anxiety in college students

The direct effect of social support on experiencing anxiety was significant, validating the idea that social support can negatively affect stress and experiencing anxiety (Beehr et al., 2010; Hou et al., 2019; Grey et al., 2020; Ma et al., 2020; Muyor-Rodríguez et al., 2021) and supporting Hypothesis 1. The overall level of college students’ anxiety during the epidemic was approximately 28.8% higher than that of the general population (Chang et al., 2020; Wang et al., 2020). The pressures of the perceived risk during COVID-19 isolation and control measures have a major impact on anxiety among college students, which is consistent with the results of previous studies (Karkoulian et al., 2016; Qiu et al., 2020).

During the period of over 2 years since December 2019, when the COVID-19 outbreak began, a scientific understanding of the virus itself has been acquired. Because the virus’ fatality rate has decreased and due to widespread vaccination against COVID-19 (WHO, 2022), the pressure induced by COVID-19 should now be lower than earlier in the outbreak. However, in this study, 35% of the respondents had anxiety scores higher than the standard value of 50 points. The reasons for the high anxiety level among college students are mainly as follows. The students perceived pressure due to COVID-19 and also had to deal with the existence of the pandemic. The high infection rate and sequelae of COVID-19 made people fearful of contracting the disease themselves or concerned about the safety of their relatives, friends, and family residing in medium- and high-risk areas. All these elements may have induced a certain degree of anxiety in the students. Additionally, the outbreak occurred at the end of the first university semester in China, during the final exam period, and thus induced anxiety in the college students already anxious because of their exams (Khan et al., 2020). Some flights and trains were canceled due to the epidemic, which resulted in plans for family reunions during the Spring Festival holiday being canceled, further increasing the college students’ anxiety. Despite being exposed to so many stressful events, when isolated college students receive support from teachers, parents, and friends, their anxiety is reduced, in turn increasing their ability to cope with stress.

### Correlations among social support, perceived risk posed by the COVID-19 epidemic, and experiencing anxiety

Considering the COVID-19 epidemic risk perception as a mediating model, this study explored how social support affects anxiety among college students through perceived risk during the university closure and isolation period. The results revealed that the students’ perceived risk had a mediating effect on the relationship between their social support and anxiety, supporting Hypothesis 2. In this study, social support significantly negatively affected perceived risk posed by COVID-19 among college students, which is consistent with the results of previous studies (Beehr et al., 2010; Wenzlaff et al., 2000). Perceived risk posed by COVID-19 significantly positively affected anxiety, which is consistent with the findings of another study (Hou et al., 2019).

Because college students may become anxious due to the risk they perceive and their plight during quarantine, social support can be beneficial in alleviating this pressure. Social support from family, friends, and teachers can ease the anxiety of college students by effectively reducing the pressure caused by the perceived risk.

In the present study, the possible mediating effects of risk perception on the correlation between social support and anxiety were investigated. Risk perception demonstrated a mediating role, which supported Hypothesis 2. Furthermore, social support exerted a significant negative effect on risk perception; this finding is consistent with those of other studies (Beehr et al., 2010; Wenzlaff et al., 2000). Moreover, risk perception exerted a significant positive effect on anxiety.

### Correlations among social support, resilience, and anxiety

Considering resilience as a mediating model, this study explored how social support affects anxiety through resilience among college students during the COVID-19 lockdown. The study results revealed that during the epidemic closure period, college students’ resilience had a mediating effect on the relationship between their social support and anxiety, supporting Hypothesis 3. Social support significantly positively affected college students’ resilience, which is consistent with the results of a previous study (Yi et al., 2005; Campbell-Sills et al., 2006; Hartley, 2008; Sheard, 2009; Yildirim and Tanriverdi, 2020), indicating that good social support can improve resilience. With social support, college students can improve their ability to rebound under pressure and cope with their current predicament under epidemic-related special circumstances (Smith et al., 2008). Additionally, resilience was discovered to significantly negatively affect anxiety, consistent with the result of another study, indicating that resilience, as a crucial indicator of anxiety and other conditions (Rosenberg, 1965), is particularly vital in the context of the COVID-19 epidemic. Resilience positively affects anxiety occurrence and development (Hou et al., 2019) and mediates the relationship between social support and experiencing anxiety (Brailovskaia et al., 2017). Therefore, family, friends, and teachers were a wellspring of powerful spiritual support that college students could access during the epidemic. While family members could provide comfort to college students only through online video and voice communication due to geographical restrictions, friends, roommates, and teachers surrounding them could communicate face-to-face with them, which greatly improved their resilience during the epidemic closure and isolation period, thereby reducing their anxiety.

College students will become high-quality talents in various industries after graduation. The public expects them to be enthusiastic, energetic, and happy. However, studies have shown that the college period is a critical period for the development of human intelligence, but it also belongs to a group with serious psychological problems (Regehr et al., 2013; Cuijpers et al., 2019; Liu et al., 2019a,b; Gao et al., 2020). According to Erikson’s (1963) theory of psychosocial development, individuals aged 19–30 belong to early adulthood, and the primary developmental task at this stage is to get along with people to develop intimacy. If developmental disabilities arise due to environmental or personal factors, individuals become socially alienated, feel lonely, and have negative emotions or psychological problems. This study found that 35% of the respondents had anxiety scores higher than the standard value of 50 points during COVID-19 isolation. However, when college students feel social support and have resilience, they can reduce anxiety during the epidemic stage. Therefore, education administrators and parents should help college students to identify the current situation of the epidemic environment, enrich relevant knowledge, and reduce perception. The negative impact and anxiety brought about by risks help them to complete their tasks in early adulthood and enable college students to make a positive connection with the social population, better equipping them to face many uncertain challenges in their future lives.

## Conclusion

In the present study, a mediation model was used to elucidate the internal impact mechanism underlying the alleviating effects of social support on the level of anxiety among college students due to campus closure because of COVID-19. The findings revealed that social support can be used to predict the level of anxiety in returning college students through the mediating effect of resilience; it can also reduce anxiety levels by controlling students’ pandemic-related risk perception. Social support, risk perception, and resilience were identified as factors affecting the level of anxiety in college students.

Because of the rollout of vaccines (WHO, 2022) and thanks to some immunity that has been naturally acquired over the past 2 years, the threat posed by COVID-19, especially the Omicron variant, has declined. Some countries such as the United Kingdom, France, Spain, Denmark, and Sweden have lifted coronavirus restrictions such as wearing a mask, implementing coronavirus passports, and restricting crowds (Anadolu Agency, 2022; BBC Chinese News, 2022). In these countries, health authorities believe that when COVID-19 becomes more predictable, it will become endemic and no longer a health emergency. However, the mandatory isolation policy has inevitably had a psychological impact on the life and study of college students. Therefore, college administrators, as service providers, should provide scientific and effective social support to students during their quarantine. For example, they should provide lectures on psychological self-regulation, raise students’ COVID-19 awareness, and strengthen personal prevention and protection. Furthermore, students can launch some activities that are permitted by the isolation and control policy. For example, they can hold professional skill competitions in dormitories, care for classmates around the dormitory, and celebrate birthdays for students with poor resilience. Moreover, counselors can attempt to increase the resilience of college students and reduce the pressure they perceive by regularly visiting the students in their dormitories to understand their life and learning difficulties; additionally, they can conduct home–school online interaction sessions and deepen the trust among college students. This would intensify the strength of social support and alleviate anxiety among college students during a COVID-19 lockdown.

Although the spread of COVID-19 can be controlled through strict control measures, such measures have severely affected the normal life and study of college students. Inevitably, college students have had to face changes including environmental shifts, and they could do only limited activities, which may have contributed to increased anxiety. Therefore, a prudent attitude should be adopted regarding quarantine measures during an outbreak such as that of COVID-19.

This study has several limitations. First, the data and related analyses were drawn from a cross-sectional design, making causal inferences is difficult. Therefore, follow-up research may integrate interviews to further supplement the research data. Second, selection bias may have occurred in this study because it was based on an online questionnaire survey, which helped prevent infection during the quarantine period for college students. In addition, because of the large sample, the model fit indices were not satisfactory, but they can be optimized in future research.

## Data availability statement

Due to confidentiality agreements, details of the data and how to request access are only available from the corresponding author, CT, upon reasonable request.

## Ethics statement

The studies involving human participants were reviewed and approved by the Scientific Research Ethics Committee of Weinan Normal University. The patients/participants provided their written informed consent to participate in this study.

## Author contributions

TH conceived of the study, participated in its design and coordination, and drafted the manuscript. CT participated in the design and coordination of the study and edited the article. XB participated in the design and helped to draft the manuscript. All authors contributed to the article and approved the submitted version.

## Funding

This study received funding from the Humanities and Social Sciences Project of the Ministry of Education of China (Research on the Early Detection of Psychological Problems and Scientific Intervention Mechanisms for College Students, no. 22JDSZ3043).

## Conflict of interest

The authors declare that the research was conducted in the absence of any commercial or financial relationships that could be construed as a potential conflict of interest.

## Publisher’s note

All claims expressed in this article are solely those of the authors and do not necessarily represent those of their affiliated organizations, or those of the publisher, the editors and the reviewers. Any product that may be evaluated in this article, or claim that may be made by its manufacturer, is not guaranteed or endorsed by the publisher.

## Footnotes

^1^ https://www.sojump.com
